# Quality assessment of functional status documentation in EHRs across different healthcare institutions

**DOI:** 10.3389/fdgth.2022.958539

**Published:** 2022-09-27

**Authors:** Sunyang Fu, Maria Vassilaki, Omar A. Ibrahim, Ronald C. Petersen, Sandeep Pagali, Jennifer St Sauver, Sungrim Moon, Liwei Wang, Jungwei W. Fan, Hongfang Liu, Sunghwan Sohn

**Affiliations:** ^1^ Department of AI and Informatics, Mayo Clinic, Rochester, MN, United States; ^2^ Department of Quantitative Health Sciences, Mayo Clinic, Rochester, MN, United States; ^3^ Department of Neurology, Mayo Clinic, Rochester, MN, United States; ^4^ Department of Medicine, Mayo Clinic, Rochester, MN, United States

**Keywords:** information quality, electronic health records, natural language processing, functional status (activity levels), aging

## Abstract

The secondary use of electronic health records (EHRs) faces challenges in the form of varying data quality-related issues. To address that, we retrospectively assessed the quality of functional status documentation in EHRs of persons participating in Mayo Clinic Study of Aging (MCSA). We used a convergent parallel design to collect quantitative and qualitative data and independently analyzed the findings. We discovered a heterogeneous documentation process, where the care practice teams, institutions, and EHR systems all play an important role in how text data is documented and organized. Four prevalent instrument-assisted documentation (iDoc) expressions were identified based on three distinct instruments: Epic smart form, questionnaire, and occupational therapy and physical therapy templates. We found strong differences in the usage, information quality (intrinsic and contextual), and naturality of language among different type of iDoc expressions. These variations can be caused by different source instruments, information providers, practice settings, care events and institutions. In addition, iDoc expressions are context specific and thus shall not be viewed and processed uniformly. We recommend conducting data quality assessment of unstructured EHR text prior to using the information.

## Introduction

The rapid adoption of electronic health record (EHR) systems has enabled the secondary use of EHR data for large-scale research discovery, real-time decision support, and data-driven workflow optimization. Unstructured text that represents a large portion of EHR data contains essential information to comprehensively represent a patient's phenotypic profile. Common types of unstructured text include clinical notes (e.g., progress, consultation, admission/discharge summary), radiology reports, pathology reports, and microbiology reports. Since the HITECH Act of 2008, there have been an increasing number of studies that use EHR text to enrich patient information in the areas of incidental findings (1), diseases and conditions with multi-factorial causes (2, 3), diseases and conditions with no singular and conclusive diagnostic tests (4), surgical information(4, 5), and social determinants of health (6). These examples strongly suggest that text information can drastically improve the discovery and detection of conditions that are not routinely coded and/or are underdiagnosed in clinical practice.

A traditional method of utilizing text from EHR systems is manual chart review, a human-assisted process of reviewing, screening, or abstracting textual information. However, this method has been criticized for being labor-intensive and time-consuming (7–10). To facilitate the large-scale secondary use of EHR text, natural language processing (NLP) has been leveraged to automatically retrieve and extract clinical information. Information retrieval for eligibility screening or cohort identification (11, 12) and information extraction for assembling clinical data sets (8, 13–18) have been widely used in EHR-based NLP applications.

Despite the promising potential, the secondary use of EHRs for developing NLP applications faces challenges in the form of varying data quality-related issues. Because EHR systems are primarily designed for patient care, the documentation of text data is affected by numerous contextual factors, including human factors, clinical environment (e.g., in-patient vs. out-patient), and practice guidelines (19–21). On one hand, the EHR system itself has a significant impact on the form and format of clinical text. The unstructured EHR text is comprised of multiple information sources such as progress reports, consultation notes, nursing flowsheets, and patient provided information. Particularly, during the information collection and documentation process, instruments such as questionnaires and templates can have a huge influence on the characteristics of clinical text. On the other hand, Built-in documentation functionality such as SmartForms (i.e., customizable text template for Epic EHR documentation), auto-population, and transcription can affect the EHR-specific syntactic and semantic definition for any data contained therein (22, 23). In our study, we define language derived from unstructured or semi-structured instruments including templates, questionnaires, assessment forms, and smart forms as instrument-assisted documentation (iDoc) expressions.

Existing studies have indicated an increasing occurrence of iDoc expressions in EHRs, which can significantly impact information redundancy and quality (24–26). However, there is a lack of systematic understanding of the data quality of these expressions as well as their downstream impact on the development, evaluation and deployment of NLP applications in single and multi-site EHR environments (19). To further investigate and quantify the text data quality issues caused by iDoc expressions and the heterogeneous EHR environments, we conducted a case study examining the availability of information on functional status focused on activities of daily living (ADL) in the EHRs of persons participating in the Mayo Clinic Study of Aging (MCSA) (27).

## Materials and methods

**Figure 1:**
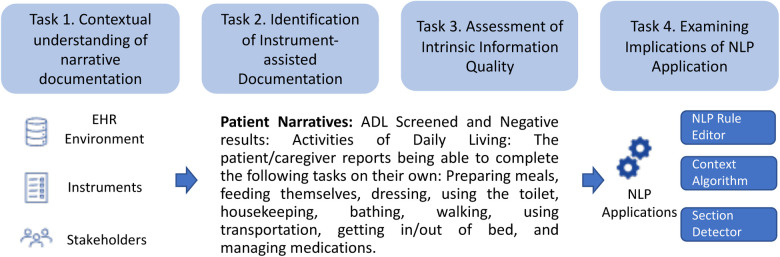
Overview of study design.

Due to the need to understand both context (e.g., how does this pattern occur?) and scope (e.g., degree and prevalence of different quality patterns), we used a convergent parallel design, a type of mixed-methods design that emphasizes concurrently collecting qualitative and quantitative data and independently analyzes findings, to assess the textual data quality across institutions. The study design is presented in Figure 1 and summarizes four sequential tasks: (1) contextual understanding of narrative documentation, (2) identification of instrument-assisted documentation, (3) assessment of information quality and (4) examining implications for NLP applications.

### Study settings

This study was approved by the Mayo Clinic and the Olmsted Medical Center Institutional Review Boards. We explored EHRs for participants from the MCSA (27), a population-based cohort study of cognitive aging with comprehensive periodic cognitive assessments, initiated in 2004. The cohort comprises 6,185 unique participants, free of dementia at the study baseline. Among these, 3,070 patients were female (49.6%) and 729 patients (11.6%) have progressed to dementia. The median age of the cohort is 73. The study cohort comprised a total of 673 patients randomly sampled from the MCSA cohort. In the sampled cohort, patients' visit times ranged from December 2004 to February 2020. Three institutions were included in the study: a tertiary care, nonprofit, and academic medical center (institution 1), a network of community-based health systems (institution 2), and a nonprofit community-based hospital (institution 3). Regarding the changes of EHR systems, Institution 1 converted its EHR system from GE Centricity to Epic in 2018, Institution 2's EHR system was converted from Cerner to Epic in 2017, and Institution 3's EHR system was converted from IC chart and Cerner to Epic in 2018.

### Contextual understanding of narrative documentation

As EHR system functionality and information documentation patterns are deeply embedded within the clinical workflow and practice, the quality of data needs to be examined for the given context (e.g., clinical setting and information documentation environment). Individual interview sessions were organized to understand the overall documentation process of patient functional status information. Interviews were conducted utilizing the snowball sampling method for recruiting participants from institution 1. Seven participants were enrolled including two geriatricians, one neurologist, three nurses, and one physical therapist. We applied contextual inquiry methods for collecting feedback. Findings were synthesized through thematic analysis.

### Identification of instrument-assisted documentation

Four consecutive steps were involved to identify the iDoc patterns: (1) identification of ADL expressions, (2) measuring textual similarity of ADL expressions, (3) manually reviewing iDoc expressions with high similarity scores, and 4) developing symbolic methods to automatically identify iDoc expressions. First, we performed corpus annotation, a task of highlighting descriptive or analytic notations applied to raw language data, to systematically identify functional status-related expressions (i.e., ADL related expressions). We used the definitions based on the International Classification of Functioning, Disability and Health (ICF) (28) to develop an annotation guideline. An iterative consensus development process was conducted involving an experienced nurse abstractor, a neurologist, three epidemiologists, and two informaticians. The annotation was performed on clinical notes. Common note types for clinical notes include visit notes, discharge summaries, physical activity reports, psychiatry reports, PT/OT consultation notes, etc., which were all included in the study. The functional status expressions related to basic activities of daily living (bADL) and instrumental activities of daily living (iADL) were manually extracted from three cohorts (randomly sampled from MCSA participants) from three institutions. Second, to identify iDoc patterns, we examined the language variation of the outputs (i.e., identified ADL expressions) from the previous step using corpus statistics and semantic textual similarity (STS) among the previously annotated ADL expressions. The measurement of STS was based on a string-matching algorithm proposed by Ratcliff and Obershelp, cosine similarity of two-word vector space, and Levenshtein distance (29–31). The method was utilized and evaluated in the 2018 BioCreative/OHNLP clinical semantic textual similarity challenge (30). A high similarity sentence pair is determined when the average score was greater or equal to 0.30. Third, based on the STS results, two trained nurse abstractors manually reviewed expressions with high similarity scores and identified iDoc-related expressions using existing questionnaire instrument forms (e.g., MC PROMIS CAT V2.0-Cognitive Function and MC PROMIS CAT V2.0 - Physical Function) as reference standards. The agreement between the two annotators was 0.907 in f1-score. Lastly, we developed symbolic methods based on manual annotation to automatically identify these iDoc expressions using an open-source information extraction system MedTaggerIE (32, 33). We then ran MedTaggerIE against the entire MCSA cohort with total of 6,185 unique patients across three sites.

### Assessment of information quality

The definitions of information quality and evaluation methods were adopted from our previous study based on the AIM quality (AIMQ) framework (34, 35). We defined three measurements for assessing intrinsic information quality (accuracy) of iDoc-related expressions of patient functional status (i.e., bADL and iADL): (1) agreement (f1-score) between two iDoc expressions that have similar semantic meaning and occur within the same clinical document (e.g., “*Have difficulty dressing: No*” vs. “*Dressing: Independent*”), (2) clinical diagnosis of mild cognitive impairment (MCI) to measure the association between iDoc functional status information and MCI diagnosis, and (3) clinical diagnosis of dementia to measure the association between iDoc functional status information and dementia diagnosis. MCI and dementia diagnoses were ascertained during the MCSA evaluations. MCI was diagnosed according to published criteria (36) and dementia was diagnosed according to DSM-IV criteria (37). For cases (patients with MCI or dementia diagnosis), clinical documents were selected prior to the index diagnosis date of MCI or dementia. For controls, there was no time constraint for document selection. The intrinsic information quality of iDoc expressions (i.e., (2) and (3)) was assessed using positive predicted value (PPV) and negative predicted value (NPV), where true positive is considered to be MCI or dementia patients with positive iDoc expressions (i.e., functional impairment) and false positive is considered to be patients with positive iDoc expressions but no formal diagnosis of MCI or dementia during the study period. Similarly, the NPV was used to examine the quality of negative iDoc expressions such as “no functional impairment”. We further quantified the contextual information (i.e., distribution of iDoc expressions) in different clinical document sections, event types, and hospital services.

### Implications for NLP applications

To understand the downstream implication of various iDoc expressions in the development, evaluation, and deployment of NLP applications for cohort-based discoveries, we summarized iDoc expressions in three syntactic representations and evaluated their impact. We ran MedTaggerIE to identify ADL-related mentions using the following scenarios: lexicon, context (ConText algorithm 38), pattern (i.e., complex rule), and section-based approach (SecTag algorithm 39). Error analysis was conducted to understand the result of each approach. We then qualitatively evaluated the implications of each iDoc expression for NLP pipelines: preprocessing pipeline, section detector, sentence detector, and context algorithm. False positive and negative cases predicted by the MedTaggerIE were manually reviewed. During the review, we manually annotated the error types including linguistics, logic, and context based on the definition from our previous review study. The process is assisted by two trained nurse abstractors (B.A.L., D.C.O.) and supervised by an informatician (S.F.).

## Results

### Contextual understanding of narrative documentation

**Figure 2:**
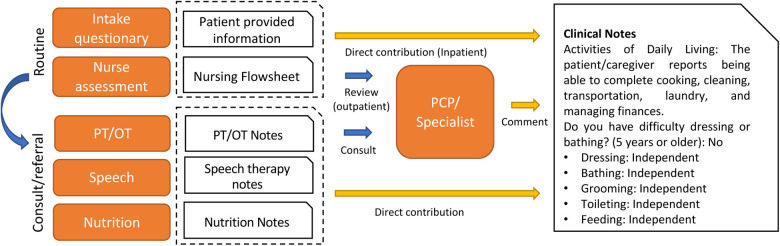
Overview of ADL documentation process at institution 1.

Figure 2 shows the overall functional status (i.e., ADL) documentation process. The qualitative thematic analysis indicates that the primary contributors to functional status documentation in clinical notes are physical and occupational therapists (PT/OT), followed by nurses, speech therapists, and nutritionists. PTs and OTs are trained to use standardized template-based documentation, smart phrases, and dictation. Findings from the PT/OT notes were based on objective measurements of functional goals. However, the documentation patterns may be heavily affected by billing practices. Nursing staff members are advised to perform routine screening to collect functional status information on the day of patients' admission. However, we learned that ADL screening and measurement can vary by care settings and patient characteristics; thus, the information may not be routinely captured. In addition, the information is stored in an independent data source (flowsheet) rather than clinical notes and indicates a need for information integration. The primary care providers (PCPs) and specialists routinely ask about patients' history of ADL information and provide only limited narrative comments. It is probable that there are also different standards and practice variations between specialists and PCPs in the evaluation and diagnosis of ADL-related conditions such as MCI and dementia.

### Identification of instrument-assisted documentation

**Figure 3:**
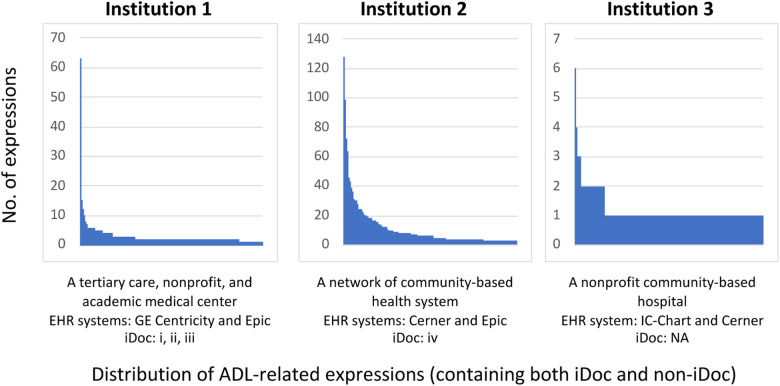
Distribution of ADL expressions in EHR documents across different institutions from the sampled annotation cohorts.

Table 1 provides the overall summary statistics of the cohort across three sites. The distribution of the unique ADL expressions from Figure 3 revealed variant patterns of frequency distribution and language variation (STS score and IQR) across three sites and suggested that institution 2 and institution 1 have more iDoc expressions than institution 3. On the other hand, institution 3 has a more descriptive and cohesive documentation style indicated by the frequency distribution (e.g., less skewed to the left). Based on the chart review, we confirmed that institution 3 had little-to-no instrument-assisted documentation patterns. Four major types of iDoc patterns were identified and presented below. The iDoc expressions i and ii are represented by a standard assertion pattern such as “able to complete” or “unable to complete” follow by inserting a standard list of events (e.g., bathing, feeding, and dressing). iDoc (iii) contains the combination of a question-and-answer format and an ADL item list. iDoc (iv) is represented by a set of standardized value sets.

**iDoc (i) Institution 1 - GE Centricity (*n* = 12,582)**:
  *Activities of Daily Living: The patient/caregiver reports being able to complete the following tasks on their own: Preparing meals, feeding themselves, dressing, using the toilet, housekeeping, bathing, walking, using transportation, getting in/out of bed, and managing medications.*
**iDoc (ii) Institution 1 - GE Centricity (*n* = 40):**
  *PATIENT NEEDS ASSISTANCE WITH THE FOLLOWING INSTRUMENTAL ACTIVITIES OF DAILY LIVING: meal preparation, medication administration, telephone use, housekeeping, shopping, managing finances, transportation use (drive car/use taxi/bus)*.
**iDoc (iii) Institution 1 – Epic (*n* = 173):**
  *Do you have serious difficulty walking or climbing stairs? Yes 04/25/2022*
  *Do you have difficulty dressing or bathing? No 04/25/2022*
  • *Dressing: Independent*
  • *Bathing: Independent*
  • *Grooming: Independent*
  • *Toileting: Independent*
  • *Feeding: Independent*
**iDoc (iv) Institution 2 – Cerner (*n* = 723):**
  *PT Goals:*
  *Bed Mobility Goal: Moderate assistance Time Frame to Reach Bed Mobility Goal: 7-day(s) Bed Mobility Goal Status: Transfer Goal: Maximal assistance Transfer Device: Other: Time Frame to Reach Transfer Goal: 7-day(s) Transfer Goal Status: Ambulation Goal: Ambulation…*

**Table 1:** Corpus statistics of three sites.

	Institution 1	Institution 2	Institution 3
No. of patients	200	203	270
No. of documents	1421	4,174	981
No. of ADL expressions	900	3,611	416
STS similarity, median (IQR)*	0.29 (0.10)	0.30 (0.17)	0.31 (0.08)

* IQR, interquartile range.

The distinct difference among different iDoc expressions suggests that they should not be uniformly viewed and processed. Some iDoc expressions can be more informative than others depending on the goal of the study. Although iDoc (i) has a high prevalence and is repetitively documented within the MCSA cohort, it can be directly used with high validity to inform patients' functional status. By contrast, iDoc (iv) is less informative without the contextual understanding of PT/OP templates and thus has limited contribution to capturing actual patients' functional status. Among four different types of iDoc expressions, we found three different syntactic representations due to the different sources (e.g., Epic smart form, questionnaire, and PT/OT templates). The question-and-answer format and PT/OT templates have a lower degree of naturality (i.e., closer to semi-structured format) than smart forms. iDoc (iii) and iDoc (iv) expressions were represented by multiple sentence fragments and posed potential challenges for sentence-level NLP models.

### Assessment of information quality and context

#### Quality assessment

Among the 714 patients with iDoc (i) expressions, the PPV for MCI and dementia are 0.735 and 0.874, respectively. Among the 3,244 patients with iDoc (ii) expressions, the NPV for non-MCI and non-dementia are 0.883 and 0.980, respectively. The agreement between two semantically similar iDoc (iii) expressions (“*Do you have difficulty dressing? No*” and “*Dressing Independent*” occurred within the same clinical document) is 0.963 in f1-score. The quality of iDoc (iv) was not assessed due to lacking a direct semantic contribution to the overall patient's functional status.

#### Context assessment

The hospital services present a higher level of heterogeneity among the four iDoc expressions. The unique number of hospital services for iDoc (i) to (iv) were 47, 183, 3, and 3 respectively. Among them, “Primary Care Internal Medicine” and “Family Medicine” were the most frequent visit types across all iDoc expressions. Regarding the event type analysis, we found 21 unique event types for iDoc (i). “Limited exam” (50%) and “Multi-system Evaluation” (38%) were the two primary events. The iDoc (ii) has 11 unique event types. The primary types include “Dismissal Summary” (76%) and “Initial Discharge Planning Assessment” (14%). Two distinct event types were found for iDoc (iii): “Progress” (81%) and “History and Physical Exam” (19%). iDoc (iv) expressions has two event types: Physical therapy progress order text (22%) and Physical therapy daily notes text (78%). The section analysis identified that iDoc (i) expressions were all based on the “System Reviews” (100%) section. We found 10 unique sections for iDoc (ii) expressions including “Instructions for continuing care” (48%), “Ongoing Care Orders” (28%), “System Reviews” (16%), and “Impression/Report/Plan” (6%). Similar to iDoc (i), iDoc (iii) expressions were uniformly based on “Social History” (98%) section. We found no sections to be mapped for iDoc (iv) expressions.

### Implications for NLP applications

In ADL expressions, the three instrument types (EHR system Smart form, Questionnaire form, and PT/OT template) showed a decreasing level of naturality of language where the smart form has the highest naturality and the PT/OP template presents a semi-structured format. The overall evaluation results of preprocessing pipeline, section detector, sentence detector, and context algorithm are illustrated in Table 2. Regarding an NLP approach to identity iDoc ADL expressions, the traditional lexicon-based approach (only using keywords) failed to identify all three instrument types. After adding the context component, the out-of-box context algorithm (assertion detection algorithm) failed to correctly determine the certainty (e.g., negated, confirmed) in the questionnaire form because the default information extraction system processes information at the sentence level. Both the questionnaire form and PT/OP template require the linkage of findings across two or more sentences to correctly determine assertion status. In addition, they also have a strong impact on both the sentence detector and context algorithm and thus require a necessary pre-processing step. By contrast, the smart form representation kept a high level of naturality of language and has minimal impact on the existing clinical information extraction pipeline (i.e., no negative impact of the section, sentence, and context algorithms). Overall, it is evident that different representations of iDoc expressions have significant implications for existing clinical information extraction applications.

**Table 2:** Summary of instrument representations and implications for NLP development.

Instrument Type	EHR system smart form	Questionnaire form	PT/OT template
Instrument Form	[Context indicator] + [Affirmative statement] + [Template elements]	[Question] + [Answer] + [Date]	[Objective] + [Description] + [Summary of assessment]
Examples	Follow up (Sec ID) PATIENT NEEDS ASSISTANCE WITH THE FOLLOWING INSTRUMENTAL ACTIVITIES OF DAILY LIVING: meal preparation, medication administration, telephone use, housekeeping, shopping, managing finances, transportation use.	Do you have serious difficulty walking or climbing stairs? Yes 04/25/2022 Do you have difficulty dressing or bathing? No 04/25/2022	PT Goals: Bed Mobility Goal: Moderate assistance Time Frame to Reach Bed Mobility Goal: 7-day (s) Bed Mobility Goal Status: Transfer Goal: Maximal assistance Transfer Device: Other: Time Frame to Reach Transfer Goal: 7-day (s) Transfer Goal Status: Ambulation Goal: Ambulation
Naturality of Language	High	Moderate	Low
Impact to Sections Detector	No	No	No
Impact to Sentence Detector	No	Yes	Yes
Impact to Context Algorithm	No	Yes	No
Preprocessing Needed for NLP Development	No	Yes	Yes

## Discussion

Our study applied a set of qualitative and quantitative methods to systematically assess the information quality of instrument-assisted documentation (iDoc expressions) across three different institutions. Four prevalent iDoc expressions were identified based on three distinct instruments: Epic smart form, questionnaire, and PT/OT templates. We found significant differences in the usage, information quality (intrinsic and contextual), and naturality of language among the different types of iDoc expressions. These variations are impacted by different source instruments, information providers, practice settings, care events, and institutions. Details in each aspect are as follows:

### Heterogeneous documentation process

The contextual inquiry revealed a dynamic, complex, and heterogeneous documentation process. Based on the interview, we discovered that the care practice teams, EHR systems, and institutions all play a significant role in how text data is documented and organized. Patient narratives are comprised of multiple different sources of information such as patients, PTs/OTs, nurses, speech therapists, nutritionists, primary care providers, and specialists. In NLP applications, information documentation and generation processes can affect the development and generalization of the models. Although the use of instruments may enhance documentation standardization, the clinician's reasoning process may be eliminated and resulted in a varying level of contextual knowledge loss. In addition, the highly variable results across three sites strongly indicated that a proper model re-training, refinement, and re-evaluation are needed.

### Semantic conflict in clinical text

The validity of clinical narratives is crucial for the secondary use of EHR data for the development of robust NLP models. Based on our intrinsic information quality assessment, we discovered a moderate-high validity of iDoc-related expressions. This finding suggests that iDoc expressions can be directly utilized for case ascertainment or common data elements in the context of this case study. However, we were able to identify conflicts amongst iDoc expressions with similar semantic meanings occurring within the same document. For example, the following expressions on the right indicate the patient's both positive and negative statuses of the ability to dress and bath: *“Do you have difficulty dressing or bathing? (5 years or older) Yes; Dressing Independent; Bathing Independent.”* We believe that such conflict may be introduced during the data collection, particularly from patient-provided questionnaires. This finding affirms that in the context of secondary use, proper data quality assessment needs to be conducted prior to the information use.

### Institutional specific iDoc documentation

The contextual information quality revealed informative contextual information for three out of four iDoc expressions. There was only a little overlap between these identified contexts among different institutions, suggesting the pattern of iDoc documentation is context specific. We also found different practices and frequencies of instrument-assisted documentation patterns across different institutions. Both institution 1 and institution 2 have heavy usage of instrument-assisted documentation for ADL mentions while institution 3 does not have iDoc expression. There is also high heterogeneity in instrument-assisted documentation between institution1 and institution 2. Our finding raises a potential issue regarding NLP algorithm portability (i.e., the ability to successfully deploy an existing NLP solution to a different environment and achieve “similar enough” results after proper system refinement) to extract ADL information from EHR text due to intrinsic documentation differences. The adoption of different instruments for patient data collection can exacerbate challenges of NLP algorithm portability, especially when using instrument-specific symbolic NLP methods. Statistics-based sub-language analysis can be considered to alleviate these challenges.

### Recommended practices for textual quality assessment

Because the quality of information is context specific, we recommend applying information quality assessments prior to any practices of information use. Four different information quality (IQ) assessments can be applied in the context of the secondary use of EHRs based on the AIMQ framework (34, 40). The framework summarized four distinct IQ dimensions: accessibility IQ, intrinsic IQ, representational IQ, and contextual IQ. These dimensions can be translated into the context of secondary use of clinical documents. The following types of quality assessment methods can be considered: clinical document accessibility (accessibility IQ), documentation completeness (contextual IQ), documentation accuracy (intrinsic IQ), and syntactic and semantic variability of clinical language (representational IQ). Clinical document accessibility can be evaluated by analyzing the information accessibility and shareability (system and method) based on different EHR settings such as intra-institution or inter-institution. In our previous study, we defined four levels of accessibility to measure the shareability of information resources across different institutions including direct access, adaptive access, partial access, and no access. Documentation completeness measures a record containing all observations made about a patient (41). Examining documentation completeness can be achieved by assessing the availability of clinical notes upon a clinical encounter. If the information is both accessible and complete, researchers can now focus on the validity of the information, which can be evaluated by comparing a particular clinical concept such as iADL with one or more reference sources. For example, the documentation of MCI-related symptoms can be found in clinical notes, nursing flowsheets, and patient-provided information. Lastly, language variability plays a significant role in NLP system portability. The form and format of clinical language can be assessed through corpus statistics and clinical textual similarity measures (35). These information quality assessments are agnostic among healthcare settings and could be applied in other settings.

### Limitations

Since we only interviewed clinicians from institution 1 due to the recruitment challenges, identified documentation practices and workflow may not be fully representative of the broader healthcare setting. In addition, since the study was conducted on three sites with a single case scenario, the generalizability of the finding is limited by the designed scope. Specifically, an institution that does not have standard guidelines and routine practices for research-level data abstraction may find it difficult to establish reference standards. In the future study, we aim to collaborate with national data consortiums such as National COVID Cohort Collaborative (N3C) and Observational Health Data Sciences and Informatics (ODHSI) to share established standard operating procedures and best practices for quality assessment. We would also like to broaden the study scope by involving more institutions and case studies.

## Conclusion

Our study applied a set of quality assessment methods to systematically examine the information quality of instrument-assisted documentation across three different institutions. We discovered a varying level of textual information documentation patterns across three institutions, the presence of semantic conflict within clinical text, and the context-specific iDoc presentations. Our study demonstrated that the quality of EHR data is closely related to the documentation workflow, stakeholders, and the functionality of individual EHR systems and thus needs to be viewed from the context of data being generated and documented. The finding affirms that data quality assessment needs to be conducted prior to the information use. These variant iDoc expressions have strong implications for NLP models. Therefore, proper model re-training, refinement, and re-evaluation are needed for a multi-institutional environment. We also discussed our recommendations for applying quality assessment methods (e.g., statistics-based sub-language analysis) for the secondary use of EHR text data. These efforts can be considered to develop and implement robust downstream applications using functional status.

## Data Availability

The datasets presented in this article are not readily available because they contain protected health information. Requests to access the datasets should be directed to the corresponding author.
